# Erysipelas-associated glomerulonephritis; a diagnostic puzzle

**Published:** 2014-07-01

**Authors:** Mohammad-Reza Ardalan

**Affiliations:** ^1^Chronic Kidney Disease Research Center, Tabriz University of Medical Sciences, Tabriz, Iran

**Keywords:** Glomerulonephritis, Infection, Erysipelas

**Implication for health policy/practice/research/medical education:**

The nephrologists, internists and general practitioners, particularly those from developing countries, should be aware of the sporadic cases of infection-related glomerulonephritis in order to diagnose and treat them early with appropriate antibiotics to prevent future renal impairment.

Infection-related glomerulonephritis (IRGN) is a newly formulated term that aims to expand our thinking beyond the post-streptococcal glomerulonephritis (PSGN) that has casted our thinking for several years. Different types of infection in different sites, mainly by immune-mediated mechanisms, could lead to glomerulonephritis (GN). During the past decades, due to increasing use of antibiotics and dramatic improvement in public hygiene, the epidemiology of infectious disease has changed dramatically, particular in developed countries. However, the epidemiology of IRGN has changed very little in most developing countries. Thus, not only the nephrologists, but also the internists and general practitioners should be aware of the sporadic cases of IRGN to diagnose them and use the appropriate antibiotic therapy early in the course of the disease to prevent future renal impairment (1,2).

A 63-year-old male was referred to nephrology clinic due to facial puffiness and malaise. There was a shiny, erythematous, edematous lesion involving eyelids, cheeks and the nose (Figure 1). On palpation, the skin was hot and tender. Pulse rate was 90/minute; temperature, 38 °C; and blood pressure, 120/90 mmHg. Laboratory examination revealed a hemoglobin of 15 g/dl, a white blood cell count of 18,700/µl, serum creatinine of 2.3 mg/dl, blood urea nitrogen of 112 mg/dl and urine analysis revealed 1+ proteinuria hematuria and 2+ hematuria with dysmorhic red blood cells in urine microscopy. Antistreptolysin O titer (ASOT) was increased to 380 IU/ml. In our patient, a renal biopsy was envisaged, however, the patient was not accepted the kidney biopsy.

**Figure 1:**
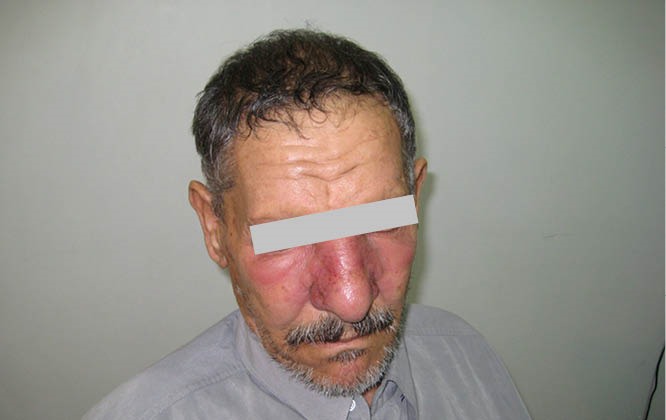
Facial puffiness and malaise. There was a shiny, erythematous, edematous lesion involving eyelids, cheeks, and the nose. (The picture is taken and published by patient permission).

## What is your diagnosis? Cellulitis!, erysipelas!, malar rash!, or angioedema?

Erysipelas is an acute dermo-hypodermal infection of bacterial origin. A distinct type of superficial cutaneous cellulitis with marked dermal lymphatic vessel involvement presenting as a painful, bright red, raised, edematous plaque, which is raised from the surrounding normal skin. It is usually caused by group A beta -hemolytic streptococcus (GAS), very uncommonly, group C or G streptococcus, and rarely due to Staphylococcus aureus. Group B streptococci can cause erysipelas in the newborn. Sometimes, there is no evident portal of entry. Sites of predilection include: face, lower legs and areas of preexisting lymphedema. The lower limbs are affected in more than 80% of the cases. In both the cellulitis and the erysipelas, the tissue feels hard on palpation and is painful, but cellulitis lesions are primarily not raised, and demarcation from uninvolved skin is indistinct. In acute urticaria and angioedema, there are superficial wheals and diffuse edema. They occur when the patient is exposed to special food or substance and there is a history of similar episodes after same exposures. There are a few reports of acute renal failure associated with erysipelas. In our patient with the primary diagnosis of erysipelas, intravenous antibiotic therapy with penicillin was started and he received appropriate fluids too. His condition improved after five days and he was discharged from the hospital. One week after discharge, his serum creatinine level was 0.8 mg/dl and urinalysis was normal.

The diagnosis of erysipelas is based upon the association of an acute inflammatory plaque with fever, lymphangitis, adenopathy and leukocytosis. Some non-infectious diseases may mimic erysipelas such as sinus venous thrombosis, familial Mediterranean fever, prosthesis intolerance (1-7).

## Author’s contribution

MRA is the single author of the paper.

## Conflict of interests

The author declared no competing interests.

## Ethical considerations

Ethical issues (including plagiarism, misconduct, data fabrication, falsification, double publication or submission, redundancy) have been completely observed by the author.

## Funding/Support

None.
